# Psychometric properties of the Chinese version of the modified polycystic ovary syndrome health-related quality-of-life questionnaire

**DOI:** 10.1186/s12955-020-01380-6

**Published:** 2020-05-11

**Authors:** Yun-yun Luo, Xiao-lin Xu, Xiao-bin Li

**Affiliations:** 1 The Second Clinical College, Guangzhou University of Chinese Medicine, Guangzhou, 510000 Guangdong China; 2 Department of Gynecology, The Second Affiliated Hospital of Guangzhou University of Traditional Chinese Medicine, Guangzhou, 510000 Guangdong China; 3 Puning hospital of traditional Chinese medicine, Puning, Jieyang, 515300 Guangdong China

**Keywords:** Polycystic ovary syndrome, Quality of life, Validation study

## Abstract

**Background:**

The modified polycystic ovary syndrome health-related quality-of-life questionnaire (MPCOSQ) is a 30-item instrument measuring quality-of-life in English-speaking patients with polycystic ovary syndrome (PCOS). We aimed to: 1) cross-culturally adapt the MPCOSQ into Chinese, and 2) assess the validity and reliability of the Chinese version of the MPCOSQ (Chi-MPCOSQ).

**Methods:**

The MPCOSQ was translated using the forward-backward method, and its validity and reliability were assessed among 283 Chinese patients with PCOS. Internal consistency reliability and test-retest reliability were assessed by Cronbach’s α and intra-correlation coefficient (ICC), respectively. Construct validity was tested through exploratory factor analysis and confirmatory factor analysis. Discriminant validity was assessed by Mann-Whitney U test to compare the scores on the Chi-MPCOSQ between 283 women with PCOS and 93 women without PCOS.

**Results:**

Exploratory factor analysis generated a 7-factor structure of the 30-item version of the Chi-MPCOSQ, which accounted for 77% of the overall variance. The Chi-MPCOSQ had high internal consistency (Cronbach’s α = 0.88) and good test-retest reliability (ICC = 0.89). Compared to PCOS patients, women without PCOS had consistently lower scores for every dimension of the Chi-MPCOSQ, demonstrating its good discriminant validity.

**Conclusion:**

The Chi-MPCOSQ is a valid and reliable instrument for measuring quality-of-life among Chinese women with PCOS.

## What is new?

1. The current study was the first one to translate the modified polycystic ovary syndrome health-related quality-of-life questionnaire (MPCOSQ) from English into Chinese and evaluate its psychometric properties among Chinese patients with polycystic ovary syndrome (PCOS).
2. Our results showed that the translated instrument had good validity and high test-retest reliability; it could also discriminant well between Chinese women with and without PCOS.
3. Our results suggested the potential clinical implication of the translated instrument in helping clinicians identify the reduced quality-of-life among Chinese women with PCOS, so that the proper interventions can be introduced to prevent associated life dissatisfaction, anxiety, and depression.

## Background

Polycystic ovary syndrome (PCOS) is a complex endocrine disorder in women of reproductive age. The prevalence varies from 5.6–16.0% among women of different ethnic groups in the world [1]. PCOS was usually associated with symptoms of emotional disturbance, weight gain, hirsutism (or body hair), acne, infertility, and menstrual irregularity [2]. These symptoms can be painful and uncomfortable and are often associated with reduced quality-of-life (QoL). Reduced QoL in PCOS is associated with life dissatisfaction, anxiety, and depression [3–6]. Therefore, assessment of QoL will provide important information for PCOS treatment, as well as intervention for and prevention of depression.

Among several PCOS-related questionnaires, the polycystic ovary syndrome health-related quality-of-life questionnaire (PCOSQ) was so far the only questionnaire directly measuring QoL in women with PCOS [7]. The instrument has been validated in a few populations and showed good reliability; however, it shows inconsistent validity due to the lack of an acne subscale [8]. Therefore, the PCOSQ was modified by Barnard et al., who added an acne subscale [2]. The modified PCOSQ (MPCOSQ) showed improved reliability and validity [2]. However, to date, the MPCOSQ has only been validated in an Iranian population [9], and it has not been validated among a Chinese population yet. Previous studies have indicated cultural and ethnic differences in the impact of PCOS on QoL; for example, compared to Caucasian women, Asian women are typically slimmer [10] and care less about body hair [11]. Thus, it is of scientific importance to validate the MPCOSQ in a Chinese population and to understand the impact of PCOS on QoL in this population.

Therefore, we aimed to cross-culturally adapt the MPCOSQ into Chinese and to evaluate the psychometric properties of the Chinese version (Chi-MPCOSQ) in Chinese patients with PCOS for the first time. Specifically, we measured the reliability (internal consistency validity and test-retest reliability) and validity (content validity and construct validity) of the Chi-MPCOSQ. Content validity measures indicates how appropriate the items of the instrument are, and construct validity evaluates how well the instrument measures the construct it was designed to measure [12]. Although validity also includes criterion validity, the latter compares the performance of the instrument with that of another instrument or predictor [13]; therefore, it was not relevant to the current study and was not included.

## Methods

### Study design and data collection

Between April 2015 and February 2016, a cross-sectional study was conducted to recruit Chinese women aged 18–45 years from the Second Affiliated Hospital of Guangzhou University of Chinese Medicine. Patients were included if they: 1) could fluently communicate in Chinese; 2) had not used any hormone, antidepressant or sedative in the previous 3 months; and 3) had been diagnosed with PCOS using the Rotterdam diagnostic criteria. In detail, the diagnosis of PCOS was made by meeting at least two of the Rotterdam diagnostic criteria: (i) polycystic ovaries visualized by an ultrasound scan (presence of 12 follicles or more in one or both ovaries and/or increased ovarian volume, i.e., 10 ml); (ii) clinical signs of hyperandrogenism (hirsutism score based on a Ferriman-Gallwey score of 0.7 or notable acne) and/or an elevated plasma testosterone level (normal testosterone level, 0.2 nmol/l); and (iii) an interval between menstrual periods of 35 days and/or amenorrhea, which was defined as the absence of vaginal bleeding for at least 6 months.

Based on medical records, we identified patients with PCOS and asked for their willingness to join the study. For those who agreed to participate, we further evaluated their eligibility. Patients were excluded if they: 1) had one of several internal medical conditions (e.g., hepatic or renal dysfunction), cerebrovascular disease, cardiovascular disease, hematologic disease, or mental illness; 2) had uncontrolled endocrine disease (e.g., thyroid disease); 3) were currently pregnant or lactating; or 4) showed poor compliance. Those who were eligible for the study and agreed to participate provided written informed consent before inclusion in the study. At recruitment, a trained interviewer conducted a face-to-face interview in the hospital for completion of the Chi-MPCOSQ and collection of information on demographic data and socioeconomic status using a structured questionnaire. Anthropometric measurements (height, weight) were measured by the interviewer. Two weeks later, the Chi-MPCOSQ was repeated among 30 patients to examine the test-retest reliability. The study protocol was approved by the Research Ethics Committee of the Second Affiliated Hospital of Guangzhou University of Chinese Medicine (IRB approval number: B2013–079-01).

### Instrument

The MPCOSQ is a specific instrument for assessing health-related QoL in PCOS patients [2]. The MPCOSQ contains 30 items (questions) with a 7-point Likert response ranging from 1 = “maximum impairment” to 7 = “no impairment” in the following seven domains: emotional disturbance (7 items), weight (6 items), hirsutism (5 items), acne (4 items), infertility (3 items), menstrual symptom (3 items), and menstrual predictability (2 items). The domain scores are the sums of the scores for the items within each domain.

### Translation of the MPCOSQ

After obtaining approval to translate the MPCOSQ into Chinese, we used the forward-backward method (Brislin’s translation model) to complete the translation [14]. First, the MPCOSQ was translated into Chinese independently by two researchers who were fluent in the Chinese language (XYL and QL). With consensus between the researchers, the draft was translated back into English by two professional translators (QL and LFH) and two medical professionals with fluent English separately (HYY and MX). Discrepancies between the translated and original questionnaires were discussed, and more than 95% of the content was the same in the two questionnaires. Adaptations were also made for the Chinese culture by a committee comprised of professionals who were fluent in both Chinese and English and were medical doctors. Subsequently, we conducted a pilot study based on cognitive interviews of 30 patients with PCOS in which the patients completed the Chi-MPCOSQ. Based on the feedback from patients, the Chi-MPCOSQ was further refined, and the final version was used to measure QoL in the current study (Supplemental Table S1).

### Statistical analysis

#### Reliability

The reliability of the Chi-MPCOSQ was assessed through internal consistency reliability, test-retest reliability and split-half reliability. Internal consistency reliability was evaluated by Cronbach’s α coefficient; test-retest reliability was evaluated by the intraclass correlation coefficient (ICC). A Cronbach’s α coefficient and a split-half coefficient ≥ 0.7 were regarded as adequate; an ICC > 0.6 indicated good agreement.

#### Validity

The validity of Chi-MPCOSQ was based on both construct validity and discriminant validity. Because the MPCOSQ was translated to Chinese for the first time and the factor structure of Chi-MPCOSQ has not been explored, exploratory factor analysis using principal component analysis with oblique promax rotation was used to examine the factor structure of the Chi-MPCOSQ [15]. The original MPCOSQ had seven domains [2]; therefore, a seven-factor solution was used for the Chi-MPCOSQ. Items with loading ≥0.40 on one factor were considered representative of that factor. To assess how well the structure extracted from the exploratory factor analysis fit the overall data, we further conducted confirmatory factor analysis [16]. Before the usage of confirmatory factor analysis, the Kaiser-Meyer-Olkin (KMO) and Bartlett’s test of sphericity statistics were applied to test the eligibility. A KMO score ≥ 0.6 indicated appropriateness for factor analysis [17]. To indicate the acceptable model fit from the confirmatory factor analysis, several indices need to meet the following criteria: 1) χ^2^/df < 5, 2) the root mean square error of approximation < 0.08, and 3) the comparative fit index > 0.9 [18]. Because the item scores were not normally distributed, discriminant validity was tested by applying the Mann-Whitney U test to compare item scores between women with and without PCOS. Six questions regarding PCOS were not applicable to women without PCOS, and thus, the scores for these questions for both groups with and without PCOS were excluded from the evaluation of discriminant validity.

#### Sample size

The recommended sample size for the exploratory factor analysis was *n* ≥ 100 or a > 2:1 ratio of subjects to items to reduce statistical errors [8]. The current study recruited 300 women with PCOS, which was more than the suggested sample size. We also recruited 100 healthy women for testing the discriminant validity of the Chi-MPCOSQ.

Statistical analysis was conducted using SPSS 17.0. Statistical significance was decided by a two-sided *P* < 0.05. Missing data for any item were replaced with the mean value for that item.

## Results

### Characteristics of recruited patients

Among the recruited 300 PCOS patients and 100 healthy women, 24 women (0.06%) were excluded due to invalid questionnaires (*n* = 16; 0.04%) or drop-out (*n* = 8; 0.02%), leaving 283 PCOS patients and 93 healthy women for the current analysis. Among the recruited subjects, no missing values existed for any variables. The characteristics of the recruited subjects are summarized in Table 1. The mean age of the PCOS patients was 25.0 (standard deviation [SD]: 4.30) years and the mean body mass index (BMI) was 22.9 (SD: 3.15) kg/m^2^. The demographics and socioeconomic status (education levels and occupation) were largely comparable between the women with and without PCOS, except that the women with PCOS were slightly heavier than those without (BMI: 22.9 [SD: 3.15] vs. 21.4 [SD: 1.72]).

**Table 1:** Characteristics of recruited subjects^a^

	Women with PCOS (*n* = 283)	Women without PCOS (*n* = 93)	*P*-value^b^
Age, years	25 (5.00)	24 (5.00)	0.97
Height, cm	158 (0.04)	158 (0.02)	0.10
Weight, kg	58 (10.0)	53 (6.00)	< 0.001
BMI, kg/m^2^	22.7 (4.52)	20.8 (2.10)	< 0.001
Education (n)			0.17
Junior high school	18 (6.36)	11 (11.8)	
High school	127 (44.9)	32 (34.4)	
University	128 (45.2)	47 (50.5)	
Graduate and above	10 (3.53)	3 (3.23)	
Occupation (n)			0.08
Medical staff	5 (1.77)	2 (2.15)	
Teachers	15 (5.30)	3 (3.23)	
Farmers	8 (2.83)	7 (7.53)	
Employees	165 (58.3)	52 (55.9)	
Student	64 (22.6)	26 (28.0)	
Unemployed	26 (9.19)	3 (3.23)	

^a^Data are presented as median (interquartile range) or n (%)

^b^*P* values based on the chi-square test for categorical variables, and Mann Whitney U test for continuous variables

### Acceptability of chi-MPCOSQ in the pilot study

In the pilot study, the mean time to finish the Chi-MPCOSQ was 10.0 (standard deviation [SD]: 3.00) minutes. The final version of the Chi-MPCOSQ was well accepted by the 30 patients in the pilot study, who said that they had no difficulties in understanding the items in the instrument.

### Chi-MPCOSQ scores in the current study

The detailed scores on the Chi-MPCOSQ for the current population are shown in Table 2. Questions regarding infertility were consistently given low scores in this population, including item 9 “Sad because of infertility problems”, item 12 “Afraid of not being able to have children”, and item 16 “Concerned about infertility problems”.

**Table 2:** Chi-MPCOSQ scores for women with and without PCOS

	Women with PCOS *(n* = 283)	Women without PCOS (n = 93)	*P*-value^b^
1. Growth of visible hair on upper lip	4.00 (3.00)	7.00 (0)	< 0.001
2. Growth of visible hair on chin	4.00 (3.00)	6.00 (0)	< 0.001
3. Growth of visible hair on face	4.00 (3.00)	7.00 (0)	< 0.001
4. Embarrassment about excessive body hair	4.00 (3.00)	6.00 (0)	< 0.001
5. Growth of visible body hair	4.00 (3.00)	7.00 (0.50)	< 0.001
6. Acne	4.00 (3.00)	7.00 (1.00)	< 0.001
7. Felt unsexy because overweight	4.00 (4.00)	6.00 (0)	< 0.001
8. Difficulties staying at your ideal weight	5.00 (3.00)	6.00 (0)	< 0.001
9. Sad because of infertility problems	4.00 (3.00)	7.00 (0)	< 0.001
10. Trouble dealing with weight	4.00 (3.00)	6.00 (0)	< 0.001
11. Frustration in trying to lose weight	5.00 (3.00)	6.00 (0)	< 0.001
12. Afraid of not being able to have children	4.00 (3.00)	6.00 (1.00)	< 0.001
13. Afraid of getting cancer	4.00 (3.00)	6.00 (0)	< 0.001
14. Concerned about being overweight	4.00 (3.00)	6.00 (0)	< 0.001
15. Easily tired	5.00 (3.00)	6.00 (1.00)	< 0.001
16. Concerned about infertility problems	4.00 (3.00)	7.00 (1.00)	< 0.001
17. Felt unattractive because of acne	5.00 (2.00)	7.00 (1.00)	< 0.001
18. Depressed as a result of acne	4.00 (3.00)	7.00 (1.00)	< 0.001
19. Lack of control over the situation with PCOS	4.00 (3.00)	–	–
20. Self-conscious as a result of having PCOS	4.00 (3.00)	–	–
21. Depressed as a result of having PCOS	4.00 (3.00)	–	–
22. Low self-esteem as a result of having PCOS	4.00 (2.00)	–	–
23. Moody as a result of having PCOS	4.00 (3.00)	–	–
24. Worried about having PCOS	4.00 (3.00)	–	–
25. Abdominal bloating^c^	4.00 (2.00)	4.00 (1.00)	< 0.001
26. Last menstruation period^c^	4.00 (2.00)	7.00 (1.00)	< 0.001
27. Menstrual cramps^c^	4.00 (3.00)	4.00 (2.50)	0.001
28. Headaches^c^	5.00 (3.00)	5.00 (2.00)	0.259
29. Irregular menstrual periods^c^	4.00 (2.00)	7.00 (1.00)	< 0.001
30. Acne^c^	4.00 (3.00)	7.00 (1.00)	< 0.001

^a^Data are presented as median (interquartile range)

^b^*P* values based on Mann-Whitney U test

^c^Refers to the last menstruation

**Abbreviations:** Chi-MPCOSQ, Chinese version of the modified polycystic ovary syndrome health-related quality-of-life questionnaire; PCOS; polycystic ovary syndrome

### Reliability

The results for reliability are shown in Table 3. For the Chi-MPCOSQ, the Cronbach’s α coefficient and ICC were 0.88 and 0.89, respectively, indicating good internal consistency reliability and test-retest reliability. High Cronbach’s α coefficients (0.69–0.95) and ICCs (0.83–0.92) were also consistently observed for the seven domains. In addition, the split-half reliability of the Chi-MPCOSQ was 0.94. In summary, the Chi-MPCOSQ showed overall good reliability.

**Table 3:** Reliability indices (Cronbach’s α coefficient, intra-class correlation coefficient, and split-half reliability) for the Chi-MPCOSQ and domains

Index	Chi-MPCOSQ	Domains						
		Hirsutism	Acne	Weight	Infertility	Emotional disturbance	Menstrual symptoms	Menstrual predictability
Cronbach’s α coefficient^a^	0.878	0.887	0.931	0.936	0.884	0.949	0.688	0.830
Intra-class correlation coefficient^b^	0.892	0.889	0.890	0.916	0.828	0.902	0.898	0.890
Split-half reliability^c^	0.938							

^a^Cronbach’s α coefficient was used to evaluate internal consistency reliability. A score ≥ 0.70 was regarded as acceptable

^b^Intra-class correlation coefficient was used to evaluate test-retest reliability. A score of > 0.60 was regarded as high agreement

^c^Split-half reliability ≥0.70 was regarded as acceptable

**Abbreviations:** Chi-MPCOSQ, Chinese version of the modified polycystic ovary syndrome health-related quality-of-life questionnaire; PCOS; polycystic ovary syndrome

### Validity

Using exploratory factor analysis with promax rotation, the Chi-MPCOSQ was found to have seven factors that explained 77.0% of the overall variance. All items loaded on one factor with loadings ranging from 0.71 to 0.92 **(**Table 4**)**, indicating good construct validity. Each individual factor explained 5.8 to 18.3% of the overall variance, and the detailed breakdown was as follows: emotional disturbance (18.3%), weight (15.7%), hirsutism (11.9%), acne (11.4%), infertility (7.56%), menstrual symptoms (6.28%), and menstrual predictability (5.85%). Consistent with the MPCOSQ, items 13 and 19–24 loaded on emotional disturbance; items 7, 8, 10, 11, 14, and 15 loaded on weight; items 1–5 loaded on hirsutism; items 6, 17, 18, and 30 loaded on acne; items 9, 12, and 16 loaded on infertility; items 25, 27, and 28 loaded on menstrual symptoms; and items 26 and 29 loaded on menstrual predictability.

**Table 4:** Seven main factors extracted by factor analysis with varimax rotation

Item	Domains						
	Emotional disturbance	Weight	Hirsutism	Acne	Infertility	Menstrual symptoms	Menstrual predictability
19. Lack of control over the situation with PCOS	0.893^a^						
20. Self-conscious as a result of having PCOS	0.880^a^						
21. Depressed as a result of having PCOS	0.874^a^						
22. Low self-esteem as a result of PCOS	0.870^a^						
23. Moody as a result of having PCOS	0.853^a^						
24. Worried about having PCOS	0.814^a^						
13. Afraid of getting cancer	0.771^a^						
7. Felt unsexy because overweight		0.887^a^					
15. Tired easily		0.873^a^					
14. Concerned about being over weight		0.861^a^					
10. Trouble dealing with weight		0.858^a^					
8. Difficulties staying at your ideal weight		0.834^a^					
11. Frustration in trying to lose weight		0.804^a^					
1. Growth of visible hair on upper lip			0.887^a^				
2. Growth of visible hair on chin			0.819^a^				
3. Growth of visible hair on your face			0.808^a^				
4. Embarrassment about excessive body hair			0.807^a^				
5. Growth of visible body hair			0.760^a^				
17. Felt unattractive because of acne				0.918^a^			
18. Depressed as a result of acne				0.912^a^			
6. Acne				0.888^a^			
30. Acne^b^				0.881^a^			
16. Concerned about infertility problems					0.851^a^		
12. Afraid of not being able to have children					0.793^a^		
9. Sad because of infertility problems					0.781^a^		
28. Headaches^b^						0.819^a^	
27. Menstrual cramps^b^						0.761^a^	
25. Abdominal bloating^b^						0.710^a^	
26. Last menstruation period^b^							0.899^a^
29. Irregular menstrual periods^b^							0.897^a^

^a^Fall into the same factor structure as the original MPCOSQ [2]

^b^Refers to the last menstruation

Abbreviations: *MPCOSQ* Modified polycystic ovary syndrome health-related quality-of-life questionnaire, *PCOS* polycystic ovary syndrome

**Fig. 1:**
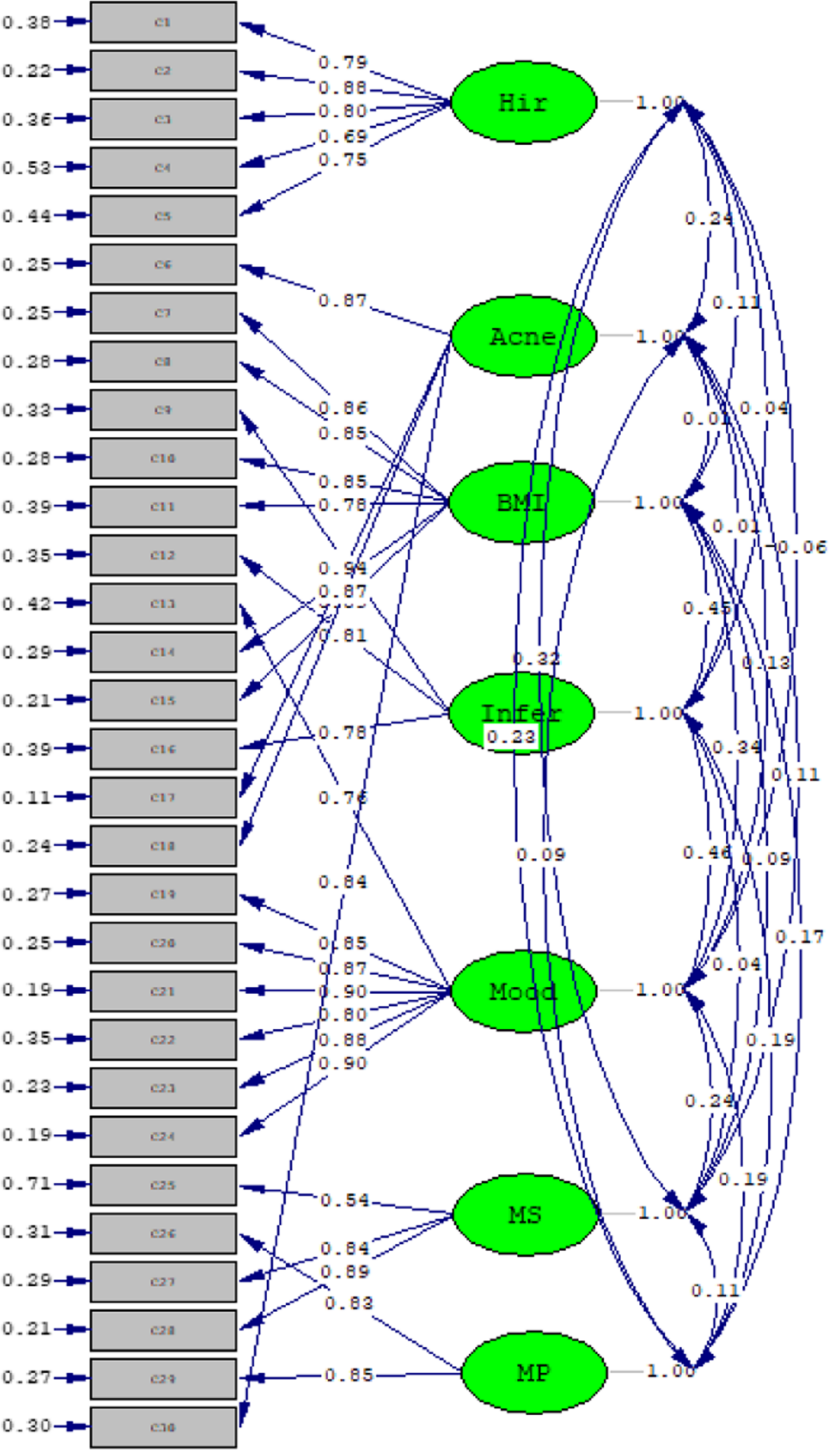
Confirmatory factor analysis of the Chi-MPCOSQ. The indices used to examine the model fitness of the seven-domain structure were as follows: χ^2^/df = 3.07, the root mean square error of approximation =0.08, and the comparative fit index =0.93. These indices suggested that the model fitness was acceptable. Abbreviations: Chi-MPCOSQ, Chinese version of the modified polycystic ovary syndrome health-related quality-of-life questionnaire; BMI, body mass index; Infer, infertility; Modd, emotional distrubance; MS, menstrual symptoms; MP, menstrual predictability.onf

The overall KMO measure of sampling adequacy was 0.83, and the result of Bartlett’s test of sphericity was significant (*P* < 0.001), indicating that confirmatory factor analysis for the current analysis was appropriate. Confirmatory factor analysis confirmed the acceptable fit of the proposed seven-domain model **(**Fig. 1**)**; specifically, 1) χ^2^/df = 4.18, 2) the root mean square error of approximation =0.08, and 3) the comparative fit index =0.93. The results for item-domain correlations of the Chi-MPCOSQ are shown in Table 5. The items had the highest Pearson correlation coefficients with their own domains, while having much weaker correlations with other domains. This result further confirmed that the seven-domain structure was appropriate for the Chi-MPCOSQ.

**Table 5:** Factor analysis and item-domain correlation analysis of the Chi-MPCOSQ

Domain	Item	Emotional disturbance	Weight	Hirsutism	Acne	Infertility	Menstrual symptoms	Menstrual predictability
Emotional disturbance	Afraid of getting cancer	0.809^a^	0.208	−0.065	0.052	0.419	0.118	0.244
	Lack of control over the situation with PCOS	0.878^a^	0.286	−0.020	0.018	0.296	0.186	0.170
	Self-conscious as a result of having PCOS	0.886^a^	0.362	−0.013	0.03	0.299	0.191	0.200
	Depressed as a result of having PCOS	0.908^a^	0.276	−0.132	0.083	0.436	0.192	0.091
	Low self-esteem as a result of having PCOS	0.838^a^	0.230	0.034	0.113	0.308	0.340	0.180
	Moody as a result of having PCOS	0.899^a^	0.240	− 0.039	0.149	0.309	0.229	0.091
	Worried about having PCOS	0.905^a^	0.326	−0.074	0.177	0.441	0.216	0.157
Weight	Felt unsexy because overweight	0.204	0.893^a^	0.081	0.039	0.328	0.021	0.127
	Difficulties staying at your ideal weight	0.339	0.872^a^	0.028	0.066	0.408	0.098	0.143
	Trouble dealing with weight	0.251	0.877^a^	0.077	0.032	0.383	0.045	0.118
	Frustration in trying to lose weight	0.286	0.834^a^	0.121	−0.040	0.324	0.094	0.125
	Concerned about being over weight	0.246	0.868^a^	0.093	−0.010	0.323	0.108	0.129
	Easily Tired	0.33	0.895^a^	0.089	0.033	0.352	0.101	0.139
Hirsutism	Growth of visible hair on upper lip	−0.071	0.127	0.826^a^	0.093	0.018	0.151	0.208
	Growth of visible hair on chin	−0.016	0.121	0.892^a^	0.122	0.017	0.212	0.195
	Growth of visible hair on face	−0.075	0.055	0.837^a^	0.258	−0.014	0.262	0.139
	Embarrassment about excessive body hair	0.005	0.022	0.774^a^	0.231	0.053	0.169	0.123
	Growth of visible body hair	−0.056	0.059	0.819^a^	0.287	0.079	0.091	0.132
Acne	Acne	0.093	0.001	0.200	0.897^a^	0.024	0.044	0.098
	Felt unattractive because of acne	0.152	−0.021	0.245	0.938^a^	− 0.001	0.112	0.095
	Depressed as a result of acne	0.046	0.009	0.202	0.913^a^	0.022	0.057	0.024
	Acne^b^	0.081	0.096	0.214	0.894^a^	0.019	0.028	0.065
Infertility	Sad because of infertility problems	0.432	0.357	0.128	0.040	0.880^a^	0.056	0.170
	Afraid of not being able to have children	0.392	0.337	0.003	−0.069	0.865^a^	0.030	0.113
	Concerned about infertility problems	0.252	0.362	−0.041	0.069	0.875^a^	−0.029	0.137
Menstrual symptoms	Abdominal bloating^b^	0.201	0.120	0	−0.026	0.031	0.725^a^	−0.079
	Menstrual cramps^b^	0.156	0.070	0.252	0.075	−0.111	0.801^a^	0.032
	Headaches^b^	0.190	0.018	0.251	0.105	0.078	0.829^a^	0.126
Menstrual predictability	Last menstruation period^b^	0.176	0.178	0.167	0.091	0.141	0.027	0.920^a^
	Irregular menstrual periods^b^	0.167	0.980	0.189	0.053	0.157	0.034	0.920^a^

^a^*P* < 0.001

^b^Refers to the last menstruation

Abbreviations: *Chi-MPCOSQ* Chinese version of the modified polycystic ovary syndrome health-related quality-of-life questionnaire, *PCOS* polycystic ovary syndrome

Discriminant validity was assessed by comparing Chi-MPCOSQ scores between women with and without PCOS **(**Tables 2 & 6**)**. The women with PCOS had significantly higher scores for 23 items (out of 24) and 6 out of 7 domains (*P* < 0.001) compared with women without PCOS, indicating worse quality of life. The domain “menstrual symptoms” had a lower median score in women with PCOS compared to those without PCOS, and the *P* value achieved a borderline significantly (*P* = 0.069). When excluding the only insignificant item “28. Headaches during menses” from the domain “menstrual symptoms”, the median value was significantly lower in in women with PCOS than those without PCOS. The results indicated that the Chi-MPCOSQ had strong discriminant validity.

**Table 6:** Discriminant validity of Chi-MPCOSQ^a^

	Women with PCOS (*n* = 283)	Women without PCOS (*n* = 93)	*P*-value^b^
Overall	131 (29.0)	188 (9.50)	< 0.001
Hirsutism	21.0 (11.0)	33.0 (1.00)	< 0.001
Acne	18.0 (9.00)	28.0 (4.00)	< 0.001
Weight	27.0 (17.0)	36.0 (2.00)	< 0.001
Infertility	12.0 (7.00)	19.0 (2.00)	< 0.001
Emotional disturbance^c^	30.0 (14.0)	48.0 (0)	< 0.001
Menstrual symptoms	14.0 (7.00)	14.0 (3.00)	0.069
Menstrual predictability	8.00 (4.00)	13.0 (2.00)	< 0.001

^a^Data are presented as median (interquartile range)

^b^*P* values based on Mann-Whitney U test

^c^Six questions regarding PCOS were not applicable to women without PCOS and were excluded from the emotional disturbance data when testing the discriminant validity of Chi-MPCOSQ

Abbreviations: *Chi-MPCOSQ* Chinese version of the modified polycystic ovary syndrome health-related quality-of-life questionnaire, *PCOS* polycystic ovary syndrome

## Discussion

To our best knowledge, the current study was the first to develop the Chi-MPCOSQ and assess its psychometric properties in Chinese women with PCOS. The Chi-MPCOSQ showed high reliability and good validity; women with and without PCOS had significantly different Chi-MPCOSQ scores, except for on one item “Headaches during menses”. Since headache during menses is not restrictive to PCOS, the instrument demonstrated good discriminant validity.

In addition to the original development study [2], the MPCOSQ so far has been validated in an Iranian population [9]. Both studies have found good reliability of the MPCOSQ (Cronbach’s α = 0.73 [2] and 0.84 [9]), corroborating the findings from the current study (Cronbach’s α = 0.88). The high reliability was also comparable with that in previous studies validating the PCOSQ [8, 11, 19, 20]]. Exploratory factor analysis is useful for exploring the number of factors of a construct [21]. Using the method, the current study found a 7-domain structure, the validity of which was further confirmed by confirmatory factor analysis and item-domain correlations. Using both exploratory and confirmatory factor analysis on the same dataset was often practiced in studies. In a review of 117 papers, 33% of the included studies applied both methods on the same sample [22]. However, some researchers were against this practice, and thought that using the same data to perform exploratory and confirmatory factor analysis will lead to overfitting in the assessment of internal structure [23, 24]. Therefore, further studies are warranted to validate the instrument structure that was explored in the current study. Although a 6-domain structure was found in the Iranian study [9], the structure of the Chi-MPCOSQ was consistent with the MPCOSQ in the original study [2]. Moreover, the percentage variance explained by the overall structure (77% vs. 80%) and individual factor (6–18% vs. 6–17%) in the current study was largely comparable to the original study [2], thus further supporting the structure validity of the Chi-MPCOSQ. Of note, the MPCOSQ had an additional acne subscale compared to the PCOSQ, which accounted for 11.4% of the overall variance in the current study. This variance was similar to the 12% in the original cohort and 10.4% in the Iranian study [2, 9]. The significant contribution of the acne subscale suggests that the MPCOSQ may more valid to use in clinical practice than the PCOSQ. The construct structure, validity and reliability need to be further validated in further studies.

In the current population, the top two concerns of women with PCOS were infertility and menstrual predictability, and these findings were consistent with the majority of previous studies where infertility and menstrual concerns were among the top concerns [2, 8, 9, 11, 20]. Interestingly, the mean score of infertility was reported to be lower in another study validating the PCOSQ among a Chinese population from Taiwan (mean: 2.38) [11]; compared to those observed in Western countries [2, 8, 20], indicating that infertility may be more of a concern in Asian countries compared to Western countries. In the current study, weight was not among the top concerns of women with PCOS; this finding was in contrast with most of the previous studies, where weight was the biggest contributor to the low QoL [2, 8, 11, 20]. The heterogeneous finding may be explained by a much lower BMI (23 kg/m^2^) in the current study compared to those of patients in studies in the UK (31–32 kg/m^2^ or 50% > 28 kg/m^2^) [2, 8], US (74% over 30 kg/m^2^) [20], and Taiwan (26.3 kg/m^2^) [11]; therefore, weight did not concern the current population as much as it did for other populations.

The Chi-MPCOSQ has important clinical implications for the treatment and management of PCOS. First, it is a useful and convenient tool (takes about 10 min to complete) to identify the causes of low QoL among women with PCOS. The identification of concerns could then facilitate targeted treatment of these women through referral to the proper medical specialist or psychological consultations that target their concerns. Second, it could also be a useful tool to evaluate the treatment effectiveness in clinical settings and research (e.g., clinical trials). Third, because PCOS is clearly associated with reduced QoL and depression [2], the identification of causes could also help with individualized intervention for depression and improvement of overall QoL and psychological health. In the validation study conducted by Griffin et al. [20], nursing was suggested to play a pivotal role in recognizing the concerns of women with PCOS and identifying approaches to support the women. Useful approaches included having online educational resources and forming support groups [20]. Thus, our findings imply that the Chi-MPCOSQ could be used in clinical settings across China.

The major strength of the current study was the large sample size. Although a ratio of 3:1 of number of participants to number of items in the instrument has been suggested to be satisfactory (from methods), a ≥ 5:1 ratio is more ideal to have adequate power for the factor analysis [25]. In the current study, we recruited 283 patients with PCOS, which corresponded to a ≥ 9:1 ratio, thus ensuring the statistical power. In addition, we included a control group to test the discriminative utility of the Chi-MPCOSQ. However, several limitations merit consideration. First, all the samples were from a single medical center in China; thus, selection bias may exist. In addition, we did not use other questionnaires in the current study. Previous studies have shown that scores on the PCOSQ or MPCOSQ correlate with those on the depression scale [2] and generic health-related QoL questionnaires such as the World Health Organization Quality of Life Questionnaire [11] and 5-dimension EuroQoL questionnaire [11]. Furthermore, the model fitness indicator of the seven-domain structure (χ2/df = 3.07) exceeded the acceptable range (3.0) suggested in previous literature [26]. Future studies are warranted to examine the structure of the scale and validate our findings.

## Conclusions

In conclusion, the Chi-MPCOSQ is a valid and reliable instrument for measuring QoL among Chinese women with PCOS. Thus, the Chi-MPCOSQ could be a useful tool to implement in clinical and research settings for the evaluation and intervention of QoL. Further studies are warranted to validate our findings in other Chinese populations.

## Supplementary information

**Additional file 1: Supplemental Table S1.** Translated version of the Chi-MPCOSQ that was used in the current study.

## Data Availability

he datasets generated and analyzed during the present study are available from the corresponding author on reasonable request.

## Abbreviations

MPCOSQ: The modified polycystic ovary syndrome health-related quality-of-life questionnaire
PCOS: Polycystic ovary syndrome
ICC: Intra-correlation coefficient
PCOSQ: Polycystic ovary syndrome health-related quality-of-life questionnaire
KMO: Kaiser-Meyer-Olkin
SD: Standard deviation
BMI: Body mass index
