# Supplementary material for: Psychometric properties of the Chinese version of the modified polycystic ovary syndrome health-related quality-of-life questionnaire
Source: Health Qual Life Outcomes. 2020 May 11;18:131. doi: 10.1186/s12955-020-01380-6 (PMC7216719; doi:10.1186/s12955-020-01380-6)
Supplement: Supplementary file 1 — Additional file 1: Supplemental Table S1. Translated version of the Chi-MPCOSQ that was used in the current study. [file 12955_2020_1380_MOESM1_ESM.docx]

**Supplemental Table S1.** Translated version of the Chi-MPCOSQ that was used in the current study.

多囊卵巢综合征患者生存质量量表

**The Modified Polycystic Ovary Syndrome Health-Related Quality-of-life Questionnaire**

本问卷询问月经和情绪的相关症状。虽然本问卷是为多囊卵巢综合征患者设计，但即使您没有患多囊卵巢综合征，我们仍希望您能填写它，因为这样能为我们提供对多囊卵巢综合征和非多囊卵巢综合征患者症状上差异的认识。请您阅读左边第一列描述， 然后选择合适的答案。

This questionnaire asks about symptoms related to menstruation and mood. It has been designed for use with women with PCOS; however, we would still like you to fill it in even if you do not have PCOS as it will give us an idea about how women with and without PCOS differ. Please read the statements in the left hand column and tick the appropriate answer.

| 在过去两周内，下面的问题对您来说属于什么程度的困扰？  To what extent were the following a problem for you during the last 2 weeks? | 非常严重的困扰  Severe problem | 严重的困扰  Major problem | 中等的困扰  Moderate problem | 有些困扰  Some problem | 一点点困扰  A small problem | 几乎沒有困扰  Hardly any problem | 完全沒困扰  No problem |
| --- | --- | --- | --- | --- | --- | --- | --- |
| 1. 您的下巴上有明显的毛发   1. Growth of visible hair on your chin |  |  |  |  |  |  |  |
| 1. 您的上唇有明显毛发   2. Growth of visible hair on your upper lip |  |  |  |  |  |  |  |
| 1. 您的脸颊有明显毛发   3. Growth of visible hair on your face |  |  |  |  |  |  |  |
| 1. 身体有明显的体毛 2. Growth of visible body hair |  |  |  |  |  |  |  |
| 1. 因体毛过多而感到尴尬   5. Feelings of embarrassment about excessive body hair |  |  |  |  |  |  |  |
| 1. 痤疮   6. Acne |  |  |  |  |  |  |  |
| 7.因为超重感到自己不够性感  7. Feel like you are not sexy because of being overweight |  |  |  |  |  |  |  |
| 8.感到很难维持在理想的体重8.Have difficulties staying at your ideal weight |  |  |  |  |  |  |  |
| 9.因为不孕问题感到伤心  9.Feel sad because of infertility problems |  |  |  |  |  |  |  |
| 10.感到很难面对自己的体重10.Had trouble dealing with your weight |  |  |  |  |  |  |  |
| 11.在努力减肥过程中感到有挫败感  11.Feet frustration in trying to lose weight |  |  |  |  |  |  |  |
| 12.害怕不能够生孩子  12.Feel afraid of not being able to have children |  |  |  |  |  |  |  |
| 13.害怕得癌症  13.Feel afraid of getting cancer |  |  |  |  |  |  |  |
| 14.担心体重超重  14.Feel concerned about being overweight |  |  |  |  |  |  |  |
| 15.容易疲倦  15.Tired easily |  |  |  |  |  |  |  |
| 1. 担心不孕的问题   16. Feel concerned about infertility problems |  |  |  |  |  |  |  |
| 1. 因为痤疮问题感到自己没有吸引力   17. Feel unattractive because of acne |  |  |  |  |  |  |  |
| 1. 因为痤疮问题感到情绪低落   18. Feel depressed as a result of acne |  |  |  |  |  |  |  |
| 19. 感觉多囊卵巢综合征的症状控制不佳  19. Feel a lack of control over the situation with PCOS |  |  |  |  |  |  |  |
| 20. 因患有多囊卵巢综合征感到情绪波动  20. Feel moody as a result of having PCOS |  |  |  |  |  |  |  |
| 21. 因患有多囊卵巢综合征感到情绪低落  21. Feel depressed as a result of having PCOS |  |  |  |  |  |  |  |
| 22. 因患有多囊卵巢综合征感到担忧  22. Feel worried about having PCOS |  |  |  |  |  |  |  |
| 23. 因患有多囊卵巢综合征感到敏感  23. Feel self-conscious as a result of having PCOS |  |  |  |  |  |  |  |
| 24. 因患有多囊卵巢综合征感到自卑  24. Experienced low self-esteem as a result of having PCOS |  |  |  |  |  |  |  |
| 25. 腹胀  25. Abdominal bloating |  |  |  |  |  |  |  |
| 26. 月经周期延后  26. Late menstrual period |  |  |  |  |  |  |  |
| 27. 痛经  27. Menstrual cramps |  |  |  |  |  |  |  |
| 28.经期头痛  28. Headaches |  |  |  |  |  |  |  |
| 29. 月经周期不规则  29. Irregular menstrual periods |  |  |  |  |  |  |  |
| 30.痤疮（上次月经）  30. Acne (last menstruation) |  |  |  |  |  |  |  |

感谢您的填写。

**Thank you for your time in completing this questionnaire**
